# Supplementary material for: MetaQTL: a package of new computational methods for the meta-analysis of QTL mapping experiments
Source: BMC Bioinformatics. 2007 Feb 8;8:49. doi: 10.1186/1471-2105-8-49 (PMC1808479; doi:10.1186/1471-2105-8-49)
Supplement: Additional File 7 — MetaQTL Package : jar file and tutorial. This Zip archive contains both the MetaQTL JAR file and the files of the tutorial. [file 1471-2105-8-49-S7.zip › org.inra.metaqtl/doc/org/metaqtl/class-use/MetaDico.html]

Uses of Class org.metaqtl.MetaDico


|  |  |  |
| --- | --- | --- |
| **Overview** \| **Package** \| **Class** \| **Use** \| **Tree** \| **Deprecated** \| **Index** \| **Help** | |  |
| PREV   NEXT | **FRAMES**    **NO FRAMES**     **All Classes** |  |


---


## **Uses of Class org.metaqtl.MetaDico**

| Packages that use MetaDico | |
| --- | --- |
| **org.metaqtl** |  |
| **org.metaqtl.algo** |  |
| **org.metaqtl.factory** |  |
| **org.metaqtl.main** |  |

Uses of MetaDico in org.metaqtl

| Methods in org.metaqtl that return MetaDico | |
| --- | --- |
| `MetaDico` | `ChromCluster.getMarkerDico()` |
| `MetaDico` | `MapCluster.getMarkerDico()` |

| Methods in org.metaqtl with parameters of type MetaDico | |
| --- | --- |
| `void` | `ChromCluster.setMarkerDico(MetaDico mrkDico)` |
| `void` | `MapCluster.setMarkerDico(MetaDico mrkDico)` |

Uses of MetaDico in org.metaqtl.algo

| Methods in org.metaqtl.algo with parameters of type MetaDico | |
| --- | --- |
| `void` | `MetaMapAlgorithm.setMarkerDico(MetaDico mrkDico)` |

Constructors in org.metaqtl.algo with parameters of type MetaDico

`MetaMapAlgorithm(IBioGenome[] maps,
IBioGenome skeleton,
IBioLocus[] dubious,
MetaDico mrkDico)`

Uses of MetaDico in org.metaqtl.factory

| Methods in org.metaqtl.factory that return MetaDico | |
| --- | --- |
| `static MetaDico` | `MetaDicoFactory.read(java.io.Reader reader)` |

Uses of MetaDico in org.metaqtl.main

| Methods in org.metaqtl.main that return MetaDico | |
| --- | --- |
| `protected static MetaDico` | `MetaMain.getDico(java.lang.String dicoFile)` |

---


|  |  |  |
| --- | --- | --- |
| **Overview** \| **Package** \| **Class** \| **Use** \| **Tree** \| **Deprecated** \| **Index** \| **Help** | |  |
| PREV   NEXT | **FRAMES**    **NO FRAMES**     **All Classes** |  |


---
